# The relationship between sarcopenia and metabolic dysfunction-associated fatty liver disease among the young and middle-aged populations

**DOI:** 10.1186/s12876-024-03192-0

**Published:** 2024-03-15

**Authors:** Ziyan Feng, Fanrong Zhao, Ziyao Wang, Xinyi Tang, Yan Xie, Li Qiu

**Affiliations:** 1 Department of Medical Ultrasound and National Clinical Research Center for Geriatrics, West China Hospital, Sichuan University, No. 37, Guoxue Lane, Wuhou District, Chengdu, Sichuan Province China; 2 Department of gastroenterology, West China Hospital, Sichuan University, No. 37, Guoxue Lane, Wuhou District, Chengdu, Sichuan Province China

**Keywords:** Sarcopenia, Metabolic dysfunction-associated fatty liver disease, Liver fibrosis, Obesity

## Abstract

**Background:**

Metabolic dysfunction-associated fatty liver disease (MAFLD) has been proposed as a new term for diagnosing fatty liver disease, which is considered to be a multi-systemic disease with multiple extrahepatic manifestations, including sarcopenia. The link between sarcopenia and MAFLD remains uncertain, especially among young and middle-aged adults. Thus, we examined the relationship between MAFLD and sarcopenia in young and middle-aged individuals in this study.

**Methods:**

A total of 2214 individuals with laboratory tests, dual-energy X-ray absorptiometry and ultrasound transient elastography from NHANES 2017–2018 were selected for this study. MAFLD was diagnosed as fatty liver disease with any one of the situations: overweight/obesity, diabetes mellitus, presence of metabolic dysregulation. Sarcopenia was defined by appendicular lean mass adjusted for body mass index (BMI). Multivariable logistic regression and restricted cubic spline (RCS) model were applied to explore the relationship between MAFLD and sarcopenia, and the mediation analyses were also conducted. Moreover, subgroup analyses stratified by BMI and lifestyles were done.

**Results:**

The prevalence of MAFLD was 47.85%, and nearly 8.05% of participants had sarcopenia. The prevalence of sarcopenia was higher in participants with MAFLD (12.75%; 95% CI 10.18–15.31%) than in the non-MAFLD (3.73%; 95% CI 2.16–5.31%). MAFLD was significantly positively associated with sarcopenia after adjustments [OR = 2.87 (95% CI: 1.62–5.09)]. Moreover, significant positive associations were observed between liver fibrosis and sarcopenia prevalence in MAFLD patients (OR = 2.16; 95% CI 1.13–4.15). The RCS curve revealed that MAFLD was linearly associated with sarcopenia. The relationship between the MAFLD and sarcopenia were mediated by C-reactive protein (mediation proportion: 15.9%) and high-density lipoprotein cholesterol (mediation proportion: 18.9%). Subgroup analyses confirmed the association between MAFLD and sarcopenia differed in different lifestyle groups.

**Conclusions:**

Both MAFLD prevalence and severity was significantly associated with sarcopenia. Thus, clinicians should advise comorbidity screening and lifestyle changes to young and middle-aged patients.

**Supplementary Information:**

The online version contains supplementary material available at 10.1186/s12876-024-03192-0.

## Background

In recent years, there has been controversy over the definition and diagnostic criteria of steatotic liver disease. The discussion mainly revolves around non-alcoholic fatty liver disease (NAFLD), metabolic dysfunction-associated fatty liver disease (MAFLD) [1], and metabolic dysfunction-associated steatotic liver disease (MASLD) [2]. Both MASLD and MAFLD are diagnosed using positive criteria, emphasizing the importance of metabolic dysfunction. A multicenter study from Brazil showed that the MAFLD definition and MASLD definition detect a similar population to NAFLD [3]. MASLD criteria are superior to MAFLD criteria in identifying fatty liver in lean patients [2]. However, MASLD is thought to potentially overdiagnose or misclassify individuals who are not at high metabolic risk. This study also reported that MAFLD performed better at detecting people who may be at greater risk for liver fibrosis and disease progression [4]. Another study demonstrated lower all-cause mortality in the MASLD-only group than in the MAFLD-only and MASLD/MAFLD overlap groups [5]. MAFLD is a liver disease with a total global prevalence of 39% [6], increasing over time and including all demographic age groups [7]. Recent studies showed that MAFLD patients had higher cardiovascular mortality than NAFLD patients [8]. NAFLD has been considered a multi-systemic disease with various extrahepatic manifestations, including non-obesity-related diseases like sarcopenia [9].

Sarcopenia is described as the gradual loss of muscle mass and strength with advancing age [10], causing a high public health burden worldwide. Given the rapid pace of population aging worldwide, the prevalence of sarcopenia is likely to grow from now on. As a progressive disorder, sarcopenia predicts adverse outcomes including frailty, disability, morbidity, and mortality [11, 12].

Sarcopenia is a multifactorial disease with contributing factors including insulin resistance, chronic inflammatory state, mitochondrial dysfunction, oxidative stress, malnutrition and inactivity [13, 14]. Key drivers for the development of MAFLD are insulin resistance, mitochondrial dysfunction, lipotoxicity, and inflammation [15], indicating that MAFLD may share common pathophysiological mechanisms with sarcopenia. A recent study focused on the influence of myosteatosis on liver stiffness in obese individuals with MAFLD [16]. Assessments of sarcopenia have been proven helpful in risk stratification among MAFLD patients [17]. However, the relationship between MAFLD and sarcopenia is unknown. Moreover, most research in this field focus on older adults. Sarcopenia is more common among older populations, but also occurs in young and middle-aged populations, as well as in early life [18–20]. Given the shift of obesity to younger populations, MAFLD is also prevalent in the young and middle-aged populations [21, 22]. Hence, it is important to detect MAFLD and sarcopenia early in life and make lifestyle changes.

Thus, we examined the relationship between MAFLD and sarcopenia in young and middle-aged adults in this study.

## Methods

### Study population

**Fig. 1:**
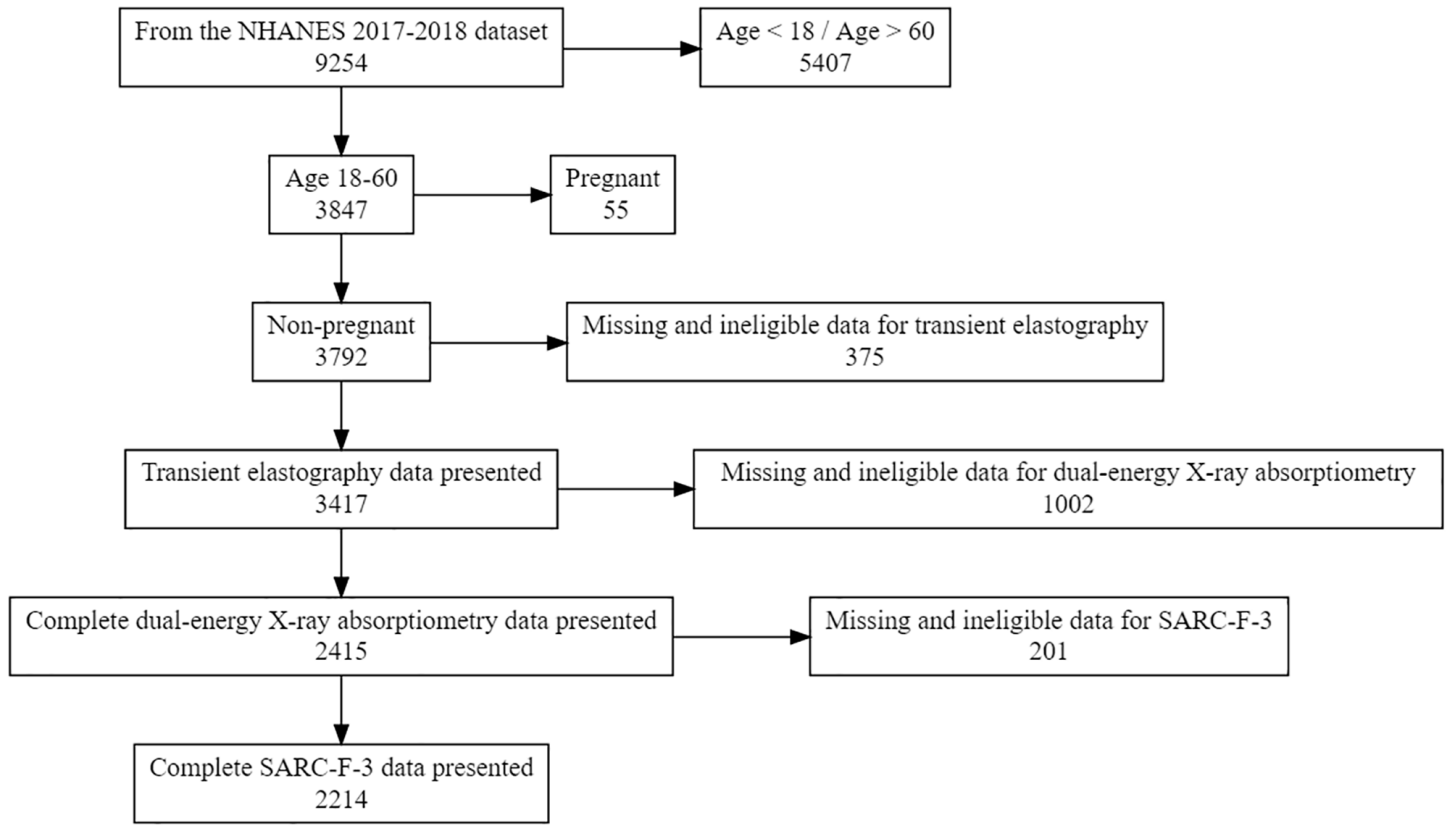
Flow chart of participants selection. Abbreviations: NHANES, National Health and Nutrition Examination Survey; SARC-F-3, SARC-F-3 (Strength, Assistance with walking, Climb stairs) questionnaire

The study utilized data from the 2017–2018 National Health and Nutrition Examination Survey (NHANES), a cross-sectional study aimed to document the US civilian non-institutionalized population. Among the 9254 participants in NHANES 2017–2018, 2214 individuals were finally enrolled in this analysis. Briefly, we excluded individuals less than 18 years old or more than 60 years old (*n* = 5407), pregnant women (*n* = 55), individuals with missing or ineligible data for transient elastography (*n* = 375), dual-energy X-ray absorptiometry (*n* = 1002), or SARC-F-3 (Strength, Assistance with walking, Climb stairs) questionnaire (*n* = 201) (Fig. 1). All participants gave informed consent, and NCHS Research Ethics Review Board approved this study (Protocol number: 2018-01).

### Definitions

MAFLD status: The diagnosis of MAFLD was based on the evidence of fatty liver disease with any one of the three situations: overweight/obesity, diabetes mellitus, or clinical evidence of metabolic dysfunction [23]. Metabolic dysfunction referred to the existence of at least two metabolic risk abnormalities: (a) Waist circumference ≥ 102 cm for men and ≥ 88 cm for women; (b) Hypertension; (c) Triglycerides (TG) ≥ 1.70 mmol/L or specific drug treatment; (d) High density lipoprotein (HDL) cholesterol < 1.0 mmol/L for men and < 1.3 mmol/L for women or specific drug treatment; (e) Pre-diabetes (fasting glucose levels 100–125 mg/dL or hemoglobin A1c 5.7–6.4%); (f) Homeostasis model assessment of insulin resistance score ≥ 2.5; (g) High-sensitivity C-reactive protein (CRP) level > 2 mg/L [24, 25]. The degree of fatty liver and liver fibrosis in NHANES 2017–2018 was evaluated using transient elastography. Median controlled attenuation parameters (CAP) ≥ 248 dB/m were selected as indicative of any steatosis [26], and CAP ≥ 331 dB/m were selected as moderate and severe steatosis [27]. CAP value within 248–331 dB/m was considered mild. In sensitivity analyses, 294 dB/m of CAP value was also utilized to define steatosis [28]. A median liver stiffness measurement (LSM) ≥ 6.3 kPa was considered as fibrosis [29]. In sensitivity analyses, 8.0 kPa of LSM value was also used to define fibrosis. Hypertension and diabetes mellitus was defined according to self-reported questionnaires. Other information was collected from laboratory tests. For sensitivity analysis, viral hepatitis patients referred to individuals with positive serum hepatitis B surface antigen or positive hepatitis C RNA. Significant alcohol consumption was also defined (men, more than three drinks per day; women, more than two drinks per day) [30].

Sarcopenia status: Appendicular lean mass (ALM) was the amount of lean mass from both arms and legs, collected through the dual-energy x-ray absorptiometry (DXA) scans. Body mass index (BMI) was estimated as weight (kg) divided by the square of the height (m). Sarcopenia was further diagnosed as ALM adjusted for BMI (< 0.789 kg/kg/m^2^ for males and < 0.512 kg/kg/m^2^ for females) [31]. SARC-F-3 is a three-item questionnaire to predict adverse outcomes of sarcopenia, based on cardinal features of sarcopenia, namely strength, assistance in walking, and climbing stairs [32], which were collected from the corresponding physical functioning questionnaire of NHANES. SARC-F-3 scores ≥ 2 were considered high scores with increased risk of sarcopenia [33].

### Covariates

From questionnaires and laboratory tests, sociodemographic covariates were obtained as potential confounders, including age (years), sex, race/ethnicity (non-Hispanic white, non-Hispanic black, Mexican American, Asian, other Hispanic, and others). In terms of lifestyle factors, alcohol intake, smoking status, and time (hours) of sedentary activity were collected. Based on the questionnaire data, participants were categorized into smokers and non-smokers. Furthermore, alcohol drinking status was categorized into non-drinkers, moderate drinkers, and heavy drinkers [23].

### Statistical analysis

Based on the analytical tutorial, we considered both the sampling design and weights of the survey [34]. We utilized the STROBE cross sectional checklist when finishing this study [35]. Multivariable logistic regression models estimated associations between MAFLD and sarcopenia, reported as odds ratios (ORs) with 95% confidence of intervals (CIs). The relationship between different degrees of MAFLD and sarcopenia, and the relationship between liver fibrosis and sarcopenia in MAFLD patients were also investigated. No further assessment is completed since the missing data for a variable was less than 10%. Multicollinearity was considered in covariates selection. Analyses were performed in a crude model without adjustment (Model 1), and then Model 2 with adjustment for sociodemographic variables. To investigate the effect size for mild steatosis compared to non-MAFLD, Model 2 was adjusted for race and sex; for moderate/severe steatosis compared to non-MAFLD, Model 2 was adjusted for race and age because of multicollinearity. Model 3 was further adjusted for alcohol consumption status and smoking status. For detecting the association between fibrosis and sarcopenia in MAFLD patients, model 3 was further adjusted only for alcohol consumption status, considering multicollinearity. Finally, Model 4 was on the basis of Model 3 and sedentary activity. To detect the relationship between MAFLD and SARC-F-3 scores, sedentary activity was not considered as covariate because of multicollinearity.

Restricted cubic spline (RCS) model was applied to explore the non-linear association. Furthermore, subgroup analyses were conducted based on BMI (< 30, ≥ 30) and different lifestyle habits including alcohol consumption status, smoking status, and sedentary activity (< 3 h, ≥ 3 h). Alcohol status was further divided into non-drinkers and drinkers. In detail, drinkers refer to those who drink one or more drinks a day, which are distinguished from non-drinkers. For mediating effect analyses, CRP, HDL and TG served as mediating variables. Each mediator model was sampled for 500 times.

All data were analyzed in R software (version 4.2.1). Statistical significance was set as *p* < 0.05.

## Results

### Study population characteristics

Based on the weighted analyses, the 2214 participants (1045 males, 49.05%; 95% CI, 46.71–51.38%) had a mean age of 39.04 (95% CI, 38.14–39.94) years. Moreover, 47.85% (95% CI: 44.65–51.06) of individuals were considered MAFLD, while the prevalence of sarcopenia reached 8.05% (95% CI: 6.20–9.89). The prevalence of sarcopenia was greater in participants with MAFLD (183 [12.75%; 95% CI, 10.18–15.31%]) than in participants without MAFLD (48 [3.73%; 95% CI, 2.16–5.31%]). The missing data for every variable was less than 10%.

Baseline characteristics of the enrolled participants were shown in Table 1. MAFLD individuals were further stratified based on the presence of liver fibrosis. Males, older adults and those with high BMI had a higher likelihood of MAFLD or liver fibrosis in MAFLD adults (all *p* < 0.05). The prevalence of smoking, malignancy and chronic kidney disease (CKD) were higher among MAFLD participants (all *p* < 0.05) and MAFLD patients with fibrosis (all *p* < 0.05). Adults without MAFLD had a higher BMI-adjusted ALM (*p* < 0.01), but the difference of BMI-adjusted ALM between MAFLD patients with and without fibrosis was not significant after stratification (*p* > 0.05). According to the sarcopenia status (defined by BMI-adjusted ALM), the baseline characteristics of the study population are compared in Table S1.

**Table 1:** Baseline characteristics of MAFLD versus non-MAFLD group and the fibrosis status of MAFLD group

Characteristics	Overall			MAFLD		
	MAFLD 47.85%^a^	Non-MAFLD 52.15%^a^	*p*-value^b^	Fibrosis 26.38%^a^	Non-Fibrosis 73.62%^a^	*p*-value^b^
**Demographic variables**						
Age (y.o.)	42.07 (0.49)	36.25 (0.69)	< 0.001	43.67 (0.89)	41.50 (0.54)	0.036
Waist circumference (cm)	107.25 (0.91)	87.61 (0.84)	< 0.001	116.55 (1.27)	103.92 (0.88)	< 0.001
BMI (kg/m^2^)	32.67 (0.37)	25.25 (0.30)	< 0.001	36.68 (0.59)	31.24 (0.35)	< 0.001
Male	53.79 (0.02)	44.77 (0.02)	< 0.001	53.55 (0.03)	53.87 (0.02)	< 0.001
Race			< 0.001			< 0.001
Non-Hispanic White	52.64 (0.04)	60.95 (0.03)		53.86 (0.05)	52.20 (0.04)	
Non-Hispanic Black	9.75 (0.02)	12.69 (0.02)		11.80 (0.03)	9.01 (0.02)	
Mexican American	15.97 (0.03)	6.98 (0.02)		15.05 (0.03)	16.30 (0.04)	
Asian	7.33 (0.02)	6.92 (0.01)		5.74 (0.01)	7.90 (0.02)	
Other Hispanic	8.79 (0.01)	8.46 (0.01)		8.78 (0.02)	8.79 (0.01)	
Other	5.53 (0.01)	3.99 (0.01)		4.76 (0.02)	5.80 (0.01)	
Smoke	41.31 (0.02)	37.64 (0.03)	< 0.001	40.76 (0.04)	41.51 (0.02)	< 0.001
Alcohol			< 0.001			< 0.001
No	22.01 (0.02)	16.26 (0.01)		23.03 (0.04)	21.64 (0.02)	
Moderate	36.57 (0.02)	35.57 (0.02)		35.55 (0.03)	36.95 (0.03)	
Heavy	41.41 (0.02)	48.17 (0.02)		41.42 (0.03)	41.41 (0.02)	
Cancer	4.68 (0.00)	4.38 (0.01)	< 0.001	4.15 (0.01)	4.87 (0.01)	0.001
Chronic kidney disease	1.51 (0.00)	1.07 (0.01)	0.017	1.23 (0.01)	1.62 (0.01)	0.015
Sedentary activity (h)	5.90 (0.21)	5.83 (0.20)	0.605	6.10 (0.30)	5.82 (0.20)	0.268
**Laboratory variables**						
GGT (IU/L)	34.77 (1.14)	23.83 (1.14)	< 0.001	43.46 (4.68)	31.63 (0.98)	0.037
Triglycerides (mmol/L)	2.04 (0.06)	1.17 (0.03)	< 0.001	2.36 (0.14)	1.93 (0.05)	0.005
ALP (IU/L)	79.35 (1.07)	70.51 (1.25)	< 0.001	81.95 (2.03)	78.41 (1.28)	0.174
AST (U/L)	23.13 (0.46)	21.71 (0.80)	0.188	27.17 (1.51)	21.68 (0.55)	0.008
ALT (U/L)	27.85 (0.60)	19.99 (0.97)	< 0.001	34.83 (1.22)	25.33 (0.82)	< 0.001
Albumin (g/dL)	4.10 (0.02)	4.19 (0.02)	< 0.001	4.05 (0.03)	4.12 (0.02)	0.019
Total bilirubin (umol/L)	7.41 (0.26)	8.56 (0.21)	0.002	7.83 (0.53)	7.26 (0.26)	0.304
CRP (mg/L)	4.58 (0.33)	2.44 (0.23)	< 0.001	4.97 (0.21)	4.44 (0.43)	0.270
HbA1c (%)	5.77 (0.06)	5.28 (0.01)	< 0.001	6.23 (0.11)	5.61 (0.05)	< 0.001
Insulin (uU/mL)	16.80 (1.12)	7.65 (0.36)	< 0.001	23.40 (1.48)	14.40 (1.03)	< 0.001
Total cholesterol (mmol/L)	5.06 (0.05)	4.71 (0.05)	< 0.001	4.99 (0.10)	5.09 (0.05)	0.365
**Sarcopenia assessment**						
ALM (g)	24,778.15 (257.09)	20,974.58 (259.96)	< 0.001	26,823.07 (399.40)	24,045.32 (369.05)	< 0.001
ALM/BMI	0.77 (0.01)	0.84 (0.01)	< 0.001	0.75 (0.01)	0.78 (0.01)	0.123

Abbreviations: BMI, body mass index; GGT, gamma-glutamyltranspeptidase; ALP, Alkaline Phosphatase; AST, aspartate aminotransferase; ALT, alanine aminotransferase; CRP, C-Reactive Protein; HbA1c, Hemoglobin A1c; ALM, appendicular lean mass

^*a*^Mean (mean.std.error); % (SE(%))

^*b*^t-test adapted to complex survey samples; Wald test of independence for complex survey samples

### Results of multivariate logistic regression analyses and restricted cubic spline model analyses

**Fig. 2:**
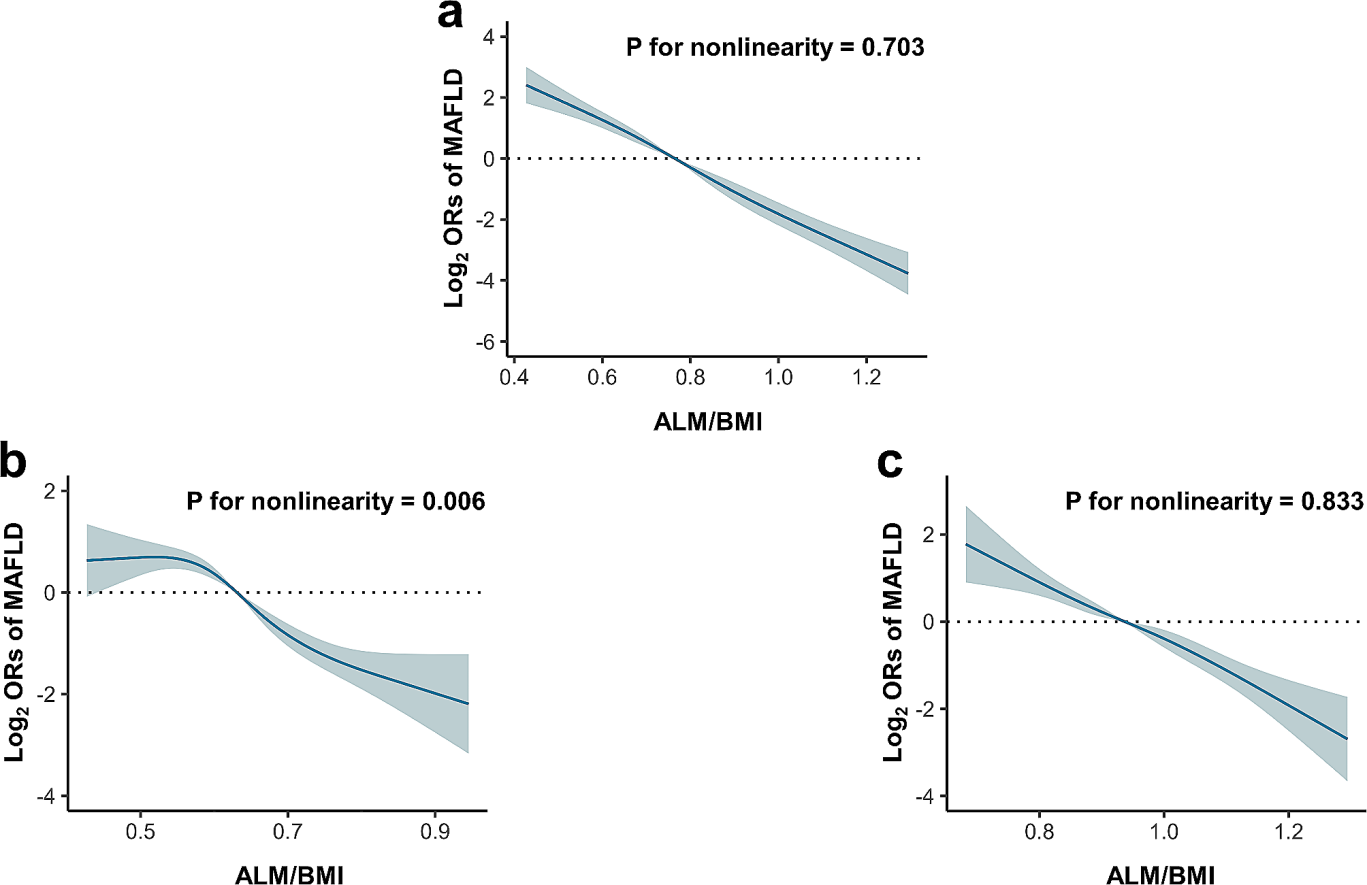
Restricted cubic spline plots of the relationship between the risk of MAFLD and ALM/BMI. (**a**) the whole research group; (**b**) women; (**c**) men. Abbreviations: BMI, body mass index; ALM, appendicular lean mass; MAFLD, metabolic dysfunction-associated fatty liver disease

The outcomes of the multivariate logistic regression analyses to examine the association between MAFLD and sarcopenia are presented in Table 2. Overall, MAFLD was significantly associated with sarcopenia in the full multivariate models [OR = 2.87 (95% CI: 1.62–5.09)]. We further subdivided the three MAFLD phenotypes: non-MAFLD, MAFLD with mild steatosis, and MAFLD with moderate and severe steatosis. MAFLD with mild steatosis was significantly associated with sarcopenia in the full multivariate models [OR = 3.45 (95% CI: 1.93–6.18)], so as the positive association between MAFLD with moderate/severe steatosis and sarcopenia [OR = 3.49 (95% CI: 1.60–7.64)]. Among the MAFLD patients, significant positive associations were observed between liver fibrosis and sarcopenia prevalence [OR = 2.16 (95% CI: 1.13–4.15)]. As for patients without significant alcohol consumption or viral hepatitis, similar relationship between MAFLD and sarcopenia was found (Table S3). For the SARC-F-3 questionnaire assessment (Table S2), no significant positive associations were observed. In Table S4, the cut-off value of steatosis was switched to 294 dB/m for sensitivity analyses. The association between MAFLD and sarcopenia still existed after adjustments [OR = 2.59 (95% CI: 1.39–4.81)]. In Table S5, the cut-off value of liver fibrosis was switched to 8.0 kPa for sensitivity analyses. The positive association between liver fibrosis and sarcopenia among the MAFLD patients still remained [OR = 3.42 (95% CI: 1.29–9.05)]. RCS models were used to assess the non-linear relationship between MAFLD and ALM/BMI (Fig. 2). No nonlinear association was found in the whole research group (*p* = 0.703 for nonlinearity) or in men (*p* = 0.833 for nonlinearity). The non-linear relationship between MAFLD and ALM/BMI was obtained in women (*p* < 0.05 for nonlinearity).

**Table 2:** The association between MAFLD and sarcopenia

	MAFLD			MAFLD Phenotypes						Fibrosis		
	OR^a^	95% CI	*p*-value	with Mild Steatosis			with Moderate and Severe Steatosis			OR^a^	95% CI	*p*-value
				OR^a^	95% CI	*p*-value	OR^a^	95% CI	*p*-value			
Model1^*b*^	3.77	2.55–5.56	< 0.001	1.06	0.81–1.39	0.638	1.32	0.87–2.02	0.179	2.18	1.30–3.64	0.006
Model2^*c*^	2.74	1.81–4.13	< 0.001	0.91	0.65–1.29	0.567	2.06	1.12–3.81	0.026	2.27	1.35–3.82	0.007
Model3^*d*^	2.82	1.71–4.63	0.004	3.36	1.97–5.73	0.002	3.36	1.59–7.13	0.009	2.17	1.19–3.95	0.021
Model4^*e*^	2.87	1.62–5.09	0.010	3.45	1.93–6.18	0.004	3.49	1.60–7.64	0.001	2.16	1.13–4.15	0.030

^*a*^OR = Odds Ratio, CI = Confidence Interval

^*b*^Crude model

^*c*^Adjusted for age, race, and sex. For MAFLD with mild steatosis, adjusted for race and sex; for MAFLD with moderate and severe steatosis, adjusted for age and race

^*d*^Further adjusted for smoking and alcohol drinking status. For fibrosis, further adjusted for alcohol drinking status

^*e*^Further adjusted for sedentary activity

### Results of subgroup analyses

**Fig. 3:**
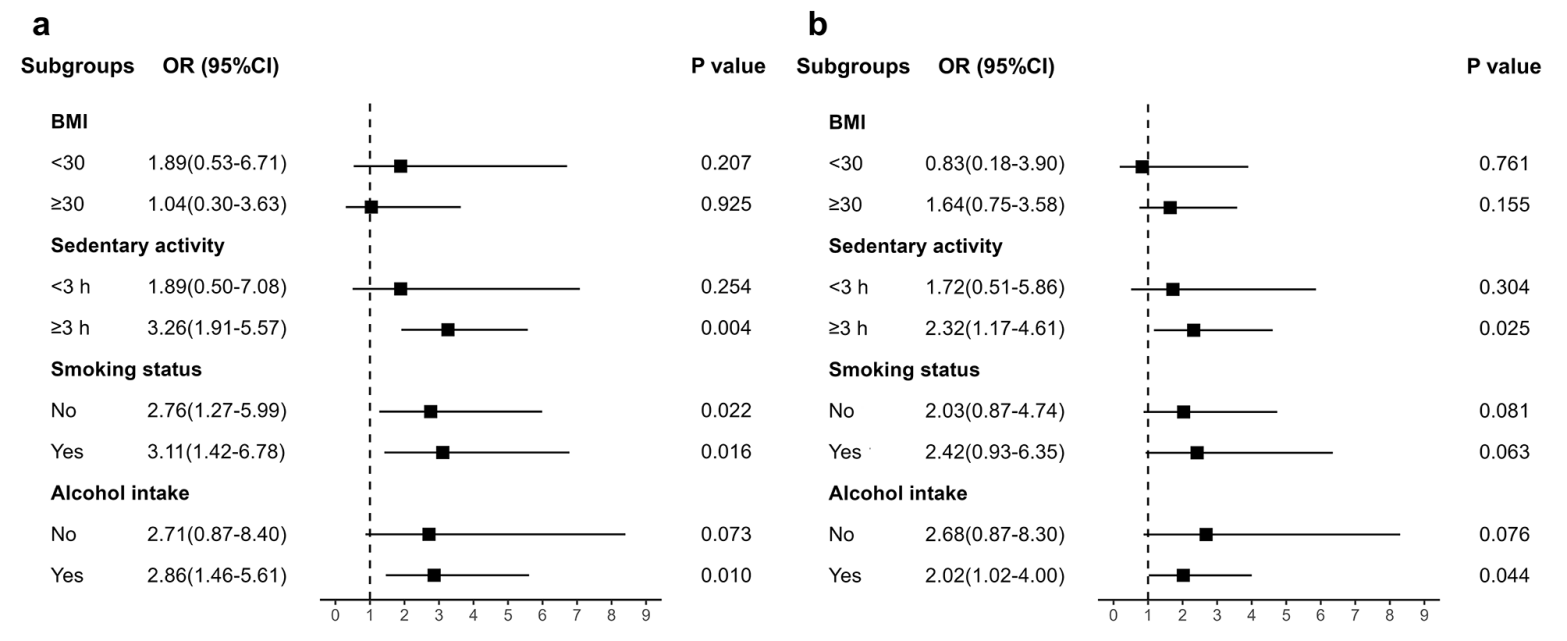
Forest plots of relationships between sarcopenia and (**a**) MAFLD, (**b**) fibrosis with subgroups. Abbreviations: BMI, body mass index; OR, Odds Ratio; CI, Confidence Interval

To examine whether lifestyles affect such relationships, subgroup analyses categorizing the participants by smoking status, alcohol drinking status, and sedentary activity were performed (Fig. 3). The association between MAFLD and sarcopenia was significant for both alcohol drinkers [OR = 2.86 (95% CI: 1.46–5.61)] and people who sit for higher than three hours per day [OR = 3.26 (95% CI: 1.91–5.57)], whereas there was no significant association in patients who have different living habits [all *p* > 0.05]. The observed association remained similar between liver fibrosis and sarcopenia among MAFLD adults. The association with MAFLD was significant for sarcopenia among both smokers and non-smokers [all *p* < 0.05], whereas the association between liver fibrosis and sarcopenia in MAFLD patients is not significant [all *p* > 0.05]. These associations were not found in the obesity subgroups defined by BMI [all *p* > 0.05].

### Results of mediation effect analyses

**Fig. 4:**
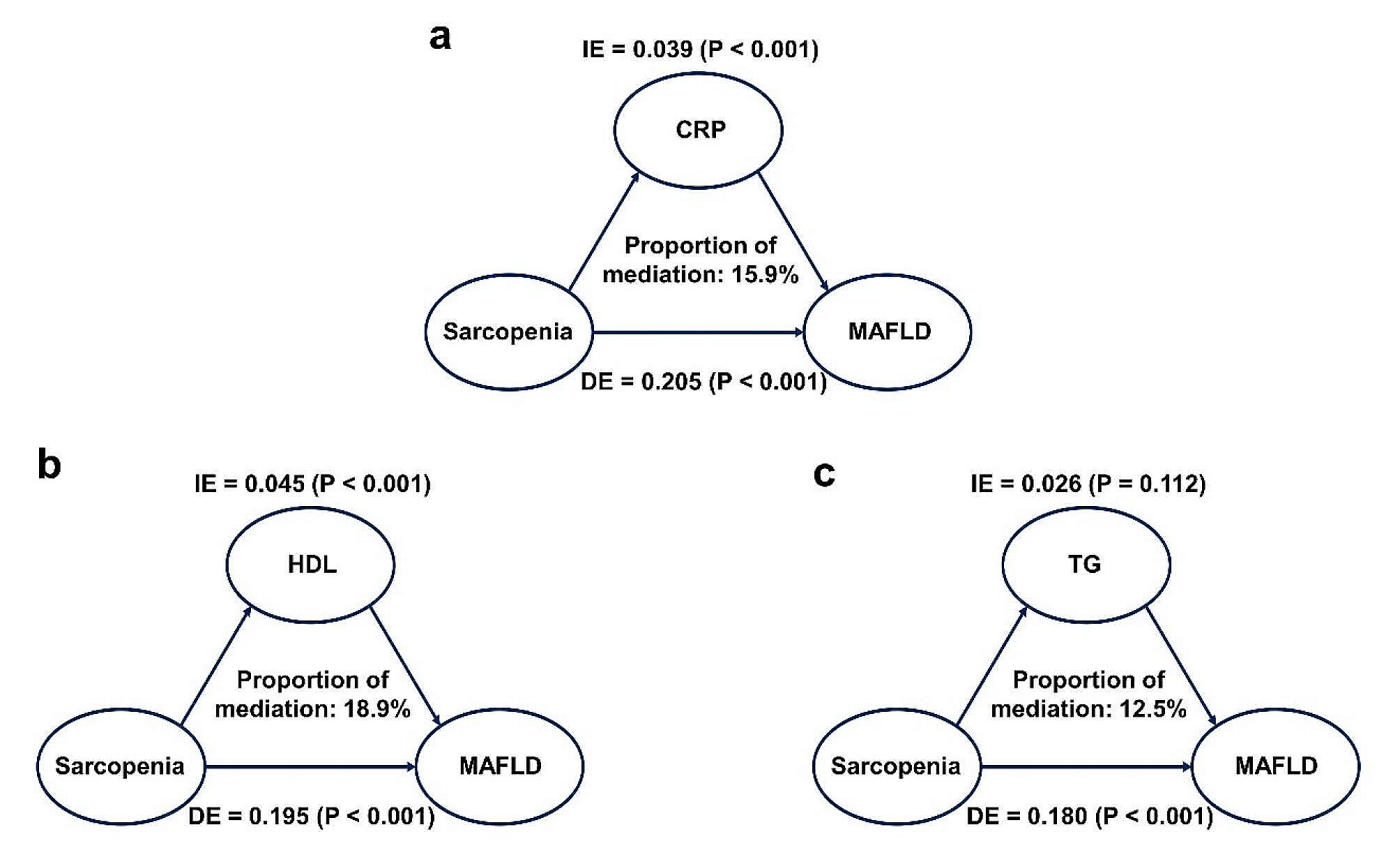
Mediation analyses of different variables on the association between MAFLD and sarcopenia. (**a**) CRP; (**b**) HDL; (**c**) TG. Abbreviations: MAFLD, metabolic dysfunction-associated fatty liver disease; CRP, C-reactive protein; HDL, high-density lipoprotein cholesterol; TG, triglycerides; IE, indirect effect; DE, direct effect

Mediation analyses were undertaken to assess if the association between the MAFLD and sarcopenia were mediated by CRP and serum lipids (Fig. 4). Direct acyclic graph for mediation analyses described the associations between sarcopenia on the MAFLD (Fig. S1). In detail, the mediated efficacy of CRP accounted for 15.9% in the associations between MAFLD and sarcopenia prevalence (IE = 0.039, 95%CI: 0.012–0.081; DE = 0.205, 95%CI: 0.134–0.272), while the mediated efficacy of HDL accounted for 18.9% (IE = 0.045, 95%CI: 0.026–0.065; DE = 0.195, 95%CI: 0.130–0.260). However, the mediation effect of TG was not significant.

## Discussion

In this representative cross-sectional study from NHANES 2017–2018, a robust association between MAFLD and sarcopenia was found in the young and middle-aged people. The association persisted after adjusting the potential covariates between MAFLD and sarcopenia, including sex, race, age, alcohol consumption status, smoking status and sedentary activity. Considering different degrees of steatosis, MAFLD phenotypes were positively associated with sarcopenia after adjustments. Among the MAFLD patients, a positive association was found between liver fibrosis and sarcopenia. Given the availability of detailed covariates and the sensitivity analyses, this study is expected to be reliable. We also found a non-linear relationship between MAFLD and sarcopenia in women, which was different from men.

Several studies have found an association between sarcopenia and NAFLD, and even advanced fibrosis [36–39]. A cohort study found that NAFLD patients were longitudinally associated with a higher risk for sarcopenia, depicted by rapid loss of skeletal muscle mass [36]. Another prospective cohort study revealed that sarcopenia was significantly related to the histological severity of NAFLD progression, proven by biopsy [37]. Insulin resistance (IR) and inflammation may contribute to this association [40]. Considering the shift to redefine NAFLD as MAFLD, more attention should be paid to this association, since MAFLD is more likely to capture patients with extrahepatic complications [41] and relate to greater fibrosis scores [6]. However, few research have assessed the association between MAFLD and sarcopenia, especially among the young and middle-aged populations. A previous research found that 7% of people aged 20–30 years have sarcopenia [42], while some recent studies found that MAFLD was not limited to the elderly [7, 43]. Similarly, our findings on the prevalence of MAFLD (47.8%, 95% CI: 44.6–51.0) and sarcopenia (8.0%, 95% CI: 6.2–9.9) further refutes the opinion that MAFLD and sarcopenia are uncommon in younger and middle-aged adults. Hence, we explored the association between MAFLD and sarcopenia, which would provide crucial suggestions for earlier disease detection and lifestyle modifications. The similar association was not observed between MAFLD and sarcopenia symptoms assessed by questionnaires. This may be because the results of the questionnaire are not as accurate as the results of the DXA scans. Since we focused on younger and middle-aged adults, the lower LSM value of 6 kPa was selected to define fibrosis. The different manifestations of different genders may be explained by differences in hormone levels, metabolic levels, and lifestyle [44–46]. Researchers have demonstrated that the relationship between low muscle strength and lean NAFLD is more pronounced in males [47]. A nonlinear relationship between MAFLD and ALM/BMI emerged in women, with a turning point in the low ALM/BMI range. This may be because women in the low ALM/BMI range have special metabolic characteristics or nutritional status. Further studies in this population are needed to explain this result.

Considering the importance of lifestyle, subgroup analyses on alcohol consumption status, smoking status and sedentary activity were conducted, to explore the possible impact of lifestyle on the relationship between MALFD and sarcopenia. Significant association was found after adjustment between MALFD and sarcopenia amongst drinkers and individuals with longer sedentary time (≥ 3 h). The relationship between liver fibrosis and sarcopenia also existed in MAFLD individuals who have these lifestyles. Even mild alcohol consumption has been considered to associate with deterioration of hepatic fibrosis among MAFLD individuals, which was consistent with our findings [48]. Associations between sedentary behavior patterns and increased risk of NAFLD have been emphasized [49, 50]. A systematic review and meta-analysis (mean or median age: 61.0–88.0 years) investigated that low sedentary behavior is linked to higher muscle strength and power [51]. Among smoking-stratified subgroups, a robust association between MAFLD and sarcopenia was also found. Our research highlights the importance of lifestyle changes in young and middle-aged populations. To prevent over-adjustment, covariates did not include obesity, but we performed a subgroup analysis on the obese populations (BMI ≥ 30). There was no association with obesity, consistent with a previous study that reported association of muscle fat content with non-alcoholic steatohepatitis instead of muscle mass [39].

We found that both MAFLD prevalence and severity was significantly associated with sarcopenia, further mediation analysis identified that CRP and HDL may mediate the impact of sarcopenia on MAFLD (*p* < 0.05). The selection of mediators and confounders was determined by searching the literature. For confounders, age, sex, race, smoking status, drinking status, and sedentary behaviors are common confounding factors when studying the sarcopenia and fatty liver [52, 53]. For mediators, multiple pathophysiological mechanisms are involved both in sarcopenia and NAFLD, including insulin resistance, chronic inflammation, cellular aging, and oxidative stress [54]. Transcription level research between NAFLD and sarcopenia have proved that the key genes for the two diseases were enriched in the lipid metabolism pathways, illustrating the importance of dyslipidemia in both diseases [55]. As for MAFLD, dyslipidemia including low HDL cholesterol levels occurs in most patients [56]. Previous studies also have shown that inflammatory cytokines are considered to be important factors in promoting the progression of MAFLD. In Chinese obese patients, high serum CRP levels were associated with an increased risk of MAFLD and were positively related to the severity of hepatic steatosis and fibrosis [57]. On the other hand, sarcopenia is thought to disrupt endocrine, metabolism and inflammation levels in the body [58]. Many studies suggest that the development of sarcopenia is related to lipid metabolism disorders [59, 60]. HDL values in patients with sarcopenia are often perturbed, with both increases and decreases seen in studies [61–63]. The mechanisms of how skeletal muscle protects lipid and lipoprotein levels are unclear and may be associated with its importance in insulin sensitivity and glycemic control [64]. Likewise, the development of chronic inflammation is important for the progression of sarcopenia, and can be reflected in circulating inflammatory cytokines [65, 66]. Relevant studies suggest that high CRP values are related with the occurrence of decreased muscle strength [67]. This study suggested a potential mediation effect of systemic inflammation and lipid metabolism abnormalities between MAFLD and sarcopenia, but the specific mechanism needs to be further studied.

This study had several limitations. First, the NHANES is a cross-sectional study, and only represent the US population. Second, NHANES utilized self-reported questionnaires for gathering variables, which might cause recall or self-reported bias. Third, liver biopsy has been considered the gold standard of fatty liver disease diagnosis, but it’s difficult to use in a large population-based survey. Hence, we used transient elastography for diagnosis [68]. Steatosis was based on CAP and fibrosis was based on LSM. Fourth, subgroup analysis of obesity explored the impact of obesity on the relationship between MAFLD and sarcopenia. The results of the subgroups with and without obesity were similar. The role of BMI in this relationship needs to be further explored. Similar results were found in other studies [69]. Finally, NHANES 2017–2018 lacks data on grip strength, which is important for assessing weakness in sarcopenia. Hence, the association of MAFLD and weakness still needs exploration.

## Conclusion

This study showed that both MAFLD prevalence and severity was significantly associated with sarcopenia among young and middle-aged individuals. Sarcopenia patients are more prone to MAFLD and MAFLD with liver fibrosis. These associations differ among people with diverse lifestyles. The relationship between the MAFLD and sarcopenia were mediated by CRP and HDL cholesterol. Thus, clinicians should advise comorbidity screening and lifestyle changes to young and middle-aged patients.

### Electronic supplementary material

Below is the link to the electronic supplementary material.

Supplementary Material 1

## Data Availability

The data that support the findings of this study are available in the National Center for Health Statistics at https://www.cdc.gov/nchs/nhanes/index.htm.

## Abbreviations

ALM: Appendicular lean mass
ALP: Alkaline phosphatase
ALT: Alanine aminotransferase
AST: Aspartate aminotransferase
BMI: Body mass index
CAP: Controlled attenuation parameter
CRP: C-reactive protein
CI: Confidence of intervals
CKD: Chronic kidney disease
CRP: C-reactive protein
DXA: Dual-energy X-ray absorptiometry
GGT: Gamma-glutamyltranspeptidase
HbA1c: Hemoglobin A1c
HDL: High-density lipoprotein
LSM: Liver stiffness measurement
MAFLD: Metabolic dysfunction-associated fatty liver disease
NAFLD: Nonalcoholic fatty liver disease
NCHS: National Center for Health Statistics
NHANES: National Health and Nutrition Examination Survey
OR: Odds ratio
SARC-F-3: (Strength, Assistance with walking, Climb stairs) questionnaire
TG: Triglycerides
US: The United States
