# Supplementary material for: The relationship between sarcopenia and metabolic dysfunction-associated fatty liver disease among the young and middle-aged populations
Source: BMC Gastroenterol. 2024 Mar 15;24:111. doi: 10.1186/s12876-024-03192-0 (PMC10943823; doi:10.1186/s12876-024-03192-0)
Supplement: Supplementary file 1 — Supplementary Material 1 [file 12876_2024_3192_MOESM1_ESM.docx]

**Table S1. Baseline characteristics of the sarcopenia group versus the non-sarcopenia group.**

| **Characteristics** | **Sarcopenia 8.05%*^a^*** | **Non-Sarcopenia 91.95%*^a^*** | ***p-value^b^*** |
| --- | --- | --- | --- |
| **Demographic variables** | | | |
| Age (y.o.) | 43.31 (1.21) | 38.70 (0.49) | 0.005 |
| Waist circumference (cm) | 111.74 (1.32) | 95.58 (0.85) | <0.001 |
| BMI (kg/m^2^) | 35.25 (0.56) | 28.18 (0.30) | <0.001 |
| Male | 57.12 (0.04) | 48.29 (0.01) | <0.001 |
| Race |  |  | <0.001 |
| Non-Hispanic White | 41.39 (0.06) | 58.48 (0.03) |  |
| Non-Hispanic Black | 3.71 (0.01) | 11.83 (0.02) |  |
| Mexican American | 27.22 (0.05) | 9.81 (0.02) |  |
| Asian | 7.73 (0.02) | 7.15 (0.01) |  |
| Other Hispanic | 13.79 (0.03) | 8.14 (0.01) |  |
| Other | 6.16 (0.03) | 4.59 (0.01) |  |
| Smoke | 40.47 (0.03) | 39.37 (0.02) | <0.001 |
| Alcohol |  |  | <0.001 |
| No | 30.25 (0.03) | 17.98 (0.01) |  |
| Moderate | 24.35 (0.05) | 37.22 (0.02) |  |
| Heavy | 45.39 (0.04) | 44.80 (0.01) |  |
| Cancer | 6.26 (0.02) | 4.41 (0.01) | 0.002 |
| Chronic kidney disease | 5.14 (0.03) | 1.01 (0.00) | 0.060 |
| Sedentary activity (h) | 4.93 (0.41) | 5.92 (0.18) | 0.021 |
| **Laboratory variables** | | | |
| Triglycerides (mmol/L) | 2.02 (0.09) | 1.55 (0.04) | <0.001 |
| CRP (mg/L) | 5.73 (0.73) | 3.26 (0.21) | 0.004 |
| HbA1c (%) | 5.91 (0.08) | 5.48 (0.03) | <0.001 |
| Insulin (uU/mL) | 20.01 (2.20) | 11.28 (0.58) | 0.001 |
| Total cholesterol (mmol/L) | 5.03 (0.09) | 4.88 (0.05) | 0.111 |
| **Sarcopenia assessment** | | | |
| ALM (g) | 22,052.49 (594.24) | 22,801.52 (217.32) | 0.299 |
| ALM/BMI | 0.63 (0.01) | 0.82 (0.01) | <0.001 |
| SARC-F-3 |  |  | 0.005 |
| <2 | 95.32 (0.02) | 98.01 (0.00) |  |
| ≥2 | 4.68 (0.02) | 1.99 (0.00) |  |
| **MAFLD assessment** | | | |
| CAP (dB/m) | 290.46 (6.90) | 252.43 (2.08) | <0.001 |
| LSM (kPa) | 8.97 (1.31) | 5.20 (0.10) | 0.013 |

Abbreviations: BMI, body mass index; CRP, C-Reactive Protein; HbA1c, Hemoglobin A1c; ALM, appendicular lean mass; SARC-F-3, SARC-F-3 (Strength, Assistance with walking, Climb stairs) questionnaire; CAP, a median Controlled Attenuation Parameter; LSM, a median Liver Stiffness Measurement
*^a^*Mean (mean.std.error); % (SE(%))
*^b^*t-test adapted to complex survey samples; Wald test of independence for complex survey samples

**Table S2. The association between MAFLD and SARC-F-3 scores.**

|  | **MAFLD** | | | **MAFLD Phenotypes** | | | | | | | | | | | | **Fibrosis** | | |
| --- | --- | --- | --- | --- | --- | --- | --- | --- | --- | --- | --- | --- | --- | --- | --- | --- | --- | --- |
|  | **OR^a^** | **95% CI** | ***p-value*** | **with Mild Steatosis** | | | | | **with Moderate and Severe Steatosis** | | | | | | | **OR^a^** | **95% CI** | ***p-value*** |
|  |  |  |  | **OR^a^** | | **95% CI** | | ***p-value*** | **OR^a^** | | | | **95% CI** | | ***p-value*** |  |  |  |
| Model1*^b^* | 1.96 | 1.19 - 3.25 | 0.012 | 1.58 | 1.04 - 2.39 | | 0.034 | | |  | 1.60 | 0.91 - 2.79 | | 0.096 | | 1.55 | 0.70 - 3.39 | 0.254 |
| Model2*^c^* | 1.77 | 1.05 - 3.00 | 0.037 | 1.31 | 0.75 - 2.30 | | 0.293 | | |  | 1.41 | 0.67 - 2.99 | | 0.315 | | 1.34 | 0.47 - 3.79 | 0.532 |
| Model3*^d^* | 1.62 | 0.87 - 3.01 | 0.096 | 1.44 | 0.72 - 2.87 | | 0.221 | | |  | 1.52 | 0.64 - 3.60 | | 0.248 | | 1.38 | 0.40 - 4.74 | 0.504 |

*^a^*OR = Odds Ratio, CI = Confidence Interval
*^b^*Crude model
*^c^*Adjusted for age, race, and sex
*^d^*Further adjusted for smoking and alcohol drinking status

**Table S3. The association between MAFLD and sarcopenia among patients without significant alcohol consumption or viral hepatitis.**

|  | **MAFLD** | | | **MAFLD Phenotypes** | | | | | | | **Fibrosis** | | |
| --- | --- | --- | --- | --- | --- | --- | --- | --- | --- | --- | --- | --- | --- |
|  | **OR***^a^* | **95% CI** | ***p-value*** | **with Mild Steatosis** | | | **with Moderate and Severe Steatosis** | | | | **OR***^a^* | **95% CI** | ***p-value*** |
|  |  |  |  | **OR***^a^* | **95% CI** | ***p-***  ***value*** | **OR***^a^* | | **95% CI** | ***p-value*** |  |  |  |
| Model1*^b^* | 4.30 | 2.53 - 7.30 | <0.001 | 3.72 | 2.03 - 6.81 | <0.001 | | 6.03 | 3.10 - 11.74 | <0.001 | 2.40 | 1.25 - 4.60 | 0.012 |
| Model2*^c^* | 3.23 | 1.67 - 6.25 | 0.004 | 3.45 | 1.65 - 7.22 | 0.005 | | 4.84 | 2.07 - 11.32 | 0.003 | 2.53 | 1.28 - 4.98 | 0.014 |
| Model3*^d^* | 3.40 | 1.55 - 7.46 | 0.012 | 3.67 | 1.55 - 8.71 | 0.012 | | 4.54 | 1.85 - 11.16 | 0.008 | 2.38 | 1.08 - 5.23 | 0.036 |
| Model4*^e^* | 3.45 | 1.39 - 8.57 | 0.023 | 3.78 | 1.50 - 9.54 | 0.016 | | 4.58 | 1.77 - 11.84 | 0.011 | 2.39 | 1.02 - 5.56 | 0.046 |

*^a^*OR = Odds Ratio, CI = Confidence Interval
*^b^*Crude model
*^c^*Adjusted for age, race and sex
*^d^*Further adjusted for smoking and alcohol drinking status. For fibrosis, further adjusted for alcohol drinking status.
*^e^*Further adjusted for sedentary activity.

**Table S4. The association between MAFLD (CAP cut-off value of 294 dB/m) and sarcopenia.**

|  | **MAFLD** | | | | **MAFLD Phenotypes** | | | | | | **Fibrosis** | | | | |
| --- | --- | --- | --- | --- | --- | --- | --- | --- | --- | --- | --- | --- | --- | --- | --- |
|  | **OR*^a^*** | **95% CI** | ***p-value*** | **with Mild Steatosis** | | | | **with Moderate and Severe Steatosis** | | | | **OR*^a^*** | **95% CI** | ***p-value*** |  |
|  |  |  |  | **OR*^a^*** | | **95% CI** | ***p-***  ***value*** | **OR*^a^*** | **95% CI** | | ***p-value*** |  |  |  |  |
| Model1*^b^* | 3.24 | 2.22 - 4.73 | <0.001 | 1.44 | | 1.09 - 1.90 | 0.014 | 1.38 | | 0.90 - 2.12 | 0.132 | 1.69 | 0.97 - 2.94 | 0.061 |  |
| Model2*^c^* | 2.47 | 1.62 - 3.76 | 0.001 | 1.22 | | 0.85 - 1.74 | 0.240 | 1.84 | | 1.03 - 3.30 | 0.042 | 1.68 | 0.91 - 3.12 | 0.087 |  |
| Model3*^d^* | 2.52 | 1.44 - 4.39 | 0.010 | 3.23 | | 1.94 - 5.40 | 0.002 | 2.62 | | 1.28 - 5.40 | 0.018 | 1.57 | 0.75 - 3.25 | 0.176 |  |
| Model4*^e^* | 2.59 | 1.39 - 4.81 | 0.016 | 3.44 | | 2.00 - 5.91 | 0.003 | 2.75 | | 1.31 - 5.76 | 0.019 | 1.55 | 0.73 - 3.31 | 0.184 |  |

*^a^*OR = Odds Ratio, CI = Confidence Interval
*^b^*Crude model
*^c^*Adjusted for age, race and sex
*^d^*Further adjusted for smoking and alcohol drinking status. For fibrosis, further adjusted for alcohol drinking status
*^e^*Further adjusted for sedentary activity

**Table S5. The association between liver fibrosis (LSM cut-off value of 8.0 kPa) and sarcopenia.**

|  | **OR*^a^*** | **95% CI** | ***p-value*** |
| --- | --- | --- | --- |
| Model1*^b^* | 3.19 | 1.51 - 6.78 | 0.005 |
| Model2*^c^* | 3.52 | 1.60 - 7.72 | 0.007 |
| Model3*^d^* | 3.44 | 1.40 - 8.46 | 0.017 |
| Model4*^e^* | 3.42 | 1.29 - 9.05 | 0.025 |
| *^a^*OR = Odds Ratio, CI = Confidence Interval *^b^*Crude model *^c^*Adjusted for age, race and sex *^d^*Further adjusted for alcohol drinking status *^e^*Further adjusted for sedentary activity | | | |


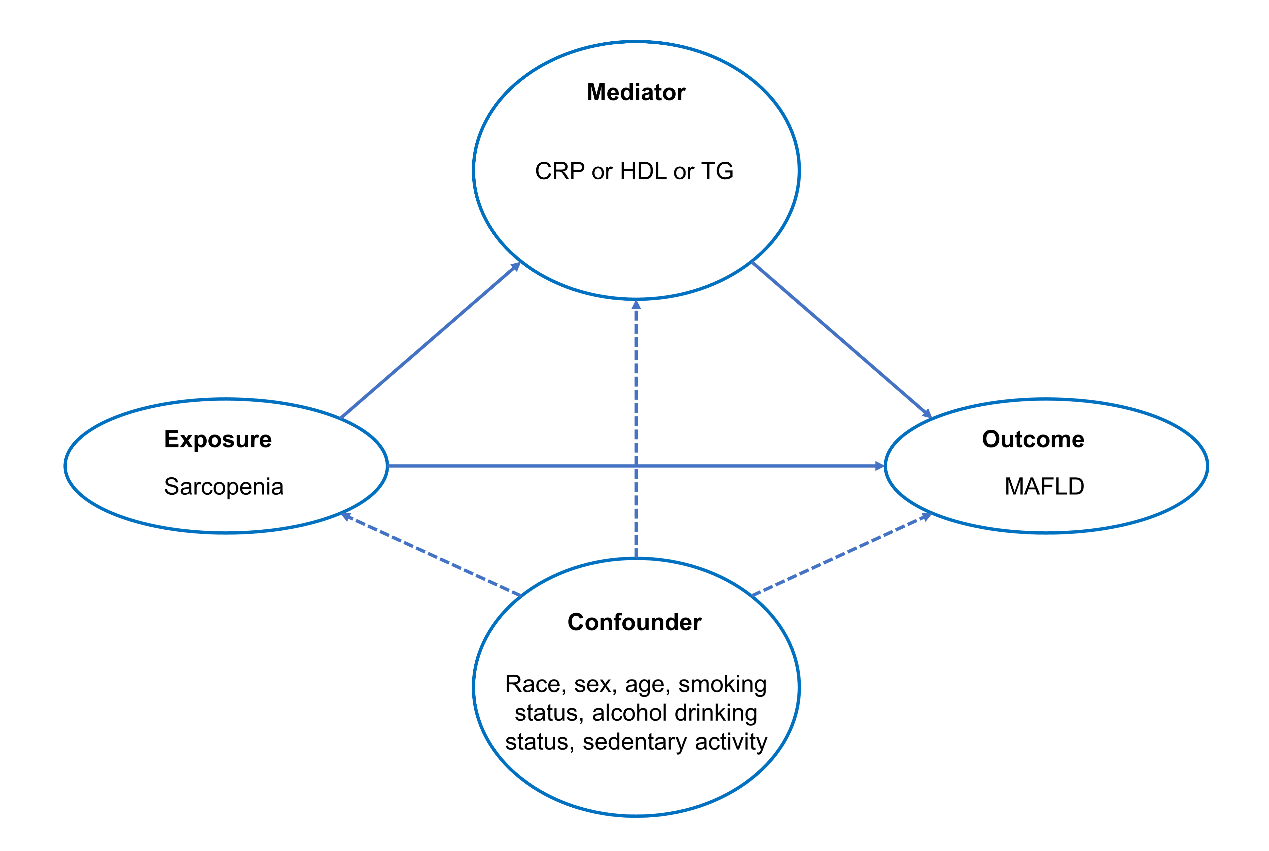


**Fig. S1** Direct acyclic graph for mediation analyses. Abbreviations: MAFLD, metabolic dysfunction-associated fatty liver disease; CRP, C-reactive protein; HDL, high-density lipoprotein cholesterol; TG, triglycerides
